# TIMELESS contributes to the progression of breast cancer through activation of MYC

**DOI:** 10.1186/s13058-017-0838-1

**Published:** 2017-05-02

**Authors:** Limin Chi, Yujiao Zou, Ling Qin, Weifeng Ma, Yanyan Hao, Yao Tang, Rongcheng Luo, Ziqing Wu

**Affiliations:** 1 Cancer Center, TCM-Integrated Hospital, Southern Medical University, Guangzhou, 510315 China; 2 Cancer Center, Traditional Chinese Medicine-Integrated Hospital, Southern Medical University, Guangzhou, 510315 China; 3 Department of Microbiology, School of Public Health, Southern Medical University, Guangzhou, 510515 China; 4 Department of Pathology, Traditional Chinese Medicine-Integrated Hospital, Southern Medical University, Shiliugang Road, Haizhu District, Guangzhou, 510315 China; 5 Key Laboratory of Molecular Tumor Pathology of Guangdong Province, Southern Medical University, Guangzhou, 510515 China; 6 Department of Pathology, School of Basic Medical Science, Southern Medical University, Guangzhou, 510515 China

**Keywords:** TIMELESS, Breast cancer, MYC, Cancer stem cells, Invasion

## Abstract

**Background:**

Breast cancer is the most common malignancy and the leading cause of cancer death among women. TIMELESS (TIM), a circadian rhythm regulator, has been recently implicated in the progression of human cancer. However, the role of TIM in the progression of breast cancer has not been well-characterized.

**Methods:**

Immunohistochemistry (IHC) staining was used to examine TIM levels in breast cancer specimens. Mammosphere formation analysis and side population analysis were used to examine the effect of TIM on the self-renewal of breast cancer stem cells. A wound healing assay and a Transwell assay were used to determine the role of TIM in breast cancer cell migration and invasion. A soft agar growth assay in vitro and tumorigenicity in vivo were used to determine the role of TIM in tumorigenicity.

**Results:**

TIM levels in both breast cancer cell lines and tissues were significantly upregulated. Patients with high TIM had poorer prognosis than patients with low TIM. Overexpression of TIM dramatically enhanced, while knockdown of TIM suppressed the self-renewal of cancer stem cells (CSCs), cell invasion and migration abilities of breast cancer cells in vitro. Moreover, overexpression of TIM significantly augmented, while knockdown of TIM reduced the tumorigenicity of breast cancer cells in vivo. Mechanism studies revealed that TIM upregulated the expression and the trans-activity of the well-known oncogene MYC. Inhibition of MYC significantly blocked the effects of TIM on CSC population, cell invasion and anchor-independent cell growth.

**Conclusion:**

TIM plays an important role in promoting breast cancer progression and may represent a novel therapeutic target for breast cancer.

**Electronic supplementary material:**

The online version of this article (doi:10.1186/s13058-017-0838-1) contains supplementary material, which is available to authorized users.

## Background

Breast cancer is the most commonly diagnosed cancer and the leading cause of cancer death among women [1]. Tumor metastasis and relapse are the main reasons for the high mortality rate in cancer. The most common cause of tumor metastasis and recurrence is the presence of cancer stem cells (CSCs), which are able to sustain self-renewal and regenerate tumors [2–4]. Thus, identification of the critical molecules that regulate self-renewal of CSCs may help to develop novel therapeutic strategies for breast cancer metastasis and recurrence.

TIMELESS (TIM) is an evolutionarily conserved protein, and is essential for circadian rhythm [5, 6]. TIM has also been demonstrated to be a replication fork-associated factor and interacts with TIM-interacting protein (Tipin) to form a stable complex and affects DNA replication [7]. For example, TIM-Tipin complex stabilizes replication forks and facilitates sister-chromatid cohesion [8]. Moreover, TIM-Tipin complex regulates DNA unwinding and DNA synthesis by directly interacting with DNA helicase and DNA polymerase (pols α, δ, and ε) and by regulating their catalytic activity [9].

Recently, TIM has been reported to be upregulated in various human tumor types and to be involved in cancer development and progression [10, 11]. In bladder cancer, TIM expression is correlated with risk of progression to muscle-invasive disease [12]. TIM is upregulated in lung cancer, and patients with high TIM expression have a poor prognosis [10]. Knockdown of TIM in hepatocellular carcinoma cells induces apoptosis, arrests cell cycle in the G2 phase, and inhibits cell migration by perturbing the interaction with eukaryotic elongation factor 1A2 (EEF1A2) [11]. Moreover, TIM is upregulated in tissue in metastatic prostate cancer, and TIM knockdown in prostate cancer cell PC3M suppresses cell migration [13]. More recently, alteration of TIM expression in breast cancer has been reported to be associated with advanced tumor stage and poorer prognosis [14, 15]. However, the precise role of TIM in the progression of breast cancer has not been fully clarified.

In the present study, we demonstrate that high expression of TIM contributes to maintenance of the CSC population, migration, invasion and tumorigenicity of breast cancer through activation of MYC. These findings reveal a key function for TIM in breast cancer progression, and may represent a novel target for breast cancer treatment.

## Methods

### Cell culture, plasmids, small interference RNAs (siRNAs), transfection and infection

Primary normal breast epithelial cell line (NBEC) was cultured in Keratinocyte serum-free medium (GIBCO) supplemented with epithelial growth factor, bovine pituitary extract. Breast cancer cell lines including BT549, MDA-MB453, MDA-MB435, SKBR3, Bcap37, MCF-7, ZR-75-30 and T47D, were obtained from the American Type Culture Collection, and grown in DMEM supplemented with 10% FBS (GIBCO).

The TIM expression construct was generated by subcloning PCR-amplified full-length human TIM cDNA into the pSin-EFα-puro vector. pSin-EFα-TIM-puro or the empty vector and two packaging plasmids pM2.G and psPAX2 were co-transfected into 293 T using the calcium phosphate transfection method, as previously described [16]. After infection, puromycin (0.5 μg/ml; Sigma) was used to select stably transducted cells. To knock down TIM and MYC, the siRNAs for TIM (siTIM) and MYC (siMYC) and their scramble control (Scramble) were purchased from RiboBio (RiboBio Inc.), and transfected using lipofectamine 2000 (Invitrogen) according to the manufacturer’s instructions.

### Human samples and immunohistochemistry (IHC) staining

A cohort of 231 paraffin-embedded human breast cancer specimens were histopathologically diagnosed at Nanfang Hospital and Traditional Chinese Medicine-Integrated Hospital, and were collected during 2000–2010. The detailed clinicopathologic characteristics of the patients with breast cancer are shown in Additional file 1: Table S1.

IHC staining was performed according to the method described previously [17]. The results of staining were evaluated and scored by two independent pathologists who were blinded to the clinical outcome, based on both the proportion of positively stained tumor cells and the intensity of staining. The proportion of tumor cells was scored as: 0 (no positive cells), 1 (less than 10% positive cells), 2 (10% to approximately 50% positive cells) and 3 (more than 50% positive cells).

The intensity of protein expression was determined as: 0 (no staining), 1 (weak staining, light yellow), 2 (moderate staining, yellowish brown) and 3 (strong staining, brown). The staining index (SI) was calculated as the product of the staining intensity and the proportion of positive cell scores (scored as 0, 1, 2, 3, 4, 6 and 9). Cutoff values for low and high expression of TIM were chosen based on a measurement of heterogeneity using the log-rank test with respect to overall survival. The optimal cutoff value was identified as follows: an SI score higher than or equal to 4 was considered to be high TIM expression and a score below 4 was considered to be low TIM expression.

### RNA isolation and real-time PCR

Total RNA was extracted using TRIzol (Invitrogen), and cDNA was synthesized using Hiscript Reverse Transcriptase (Vazyme) according to manufacturer instructions. Real-time PCR was performed in triplicate using AceQ qPCR SYBR Green Master Mix (Vazyme) on the CFX-96 Real-time PCR Detection System (Bio-Rad). Glyceraldehyde-3-phosphate dehydrogenase (GAPDH) was used as an endogenous normalization control. The cycle threshold (Ct) value was used for quantification using the 2^-ΔΔCt^ method. Sequences of the primers are: *TIM*, forward: 5′-CTCCTCCGGGCTTCTGA-3′; reverse: 5′-CCATACATCAGTGGACCAACC-3′; *GAPDH,* forward: 5′-GACTCATGACCACAGTCCATGC-3′; reverse: 5′-AGAGGCAGGGATGATGTTCTG-3′.

#### Western blot

Western blot was performed according to a previous method [18]. The primary antibodies were shown as follows: anti-TIM (Abcam), anti-MYC (Cell Signaling, Danvers, MA, USA). The membranes were stripped and re-probed with an anti-β-actin as the loading control.

### Mammosphere formation analysis

Cells (200/well) were plated on 48-well ultra-low-attachment plates (Fisher) grown in serum-free DMEM-F12 (GIBCO) supplemented with B27, 20 ng/ml epidermal growth factor (EGF) (BD), 0.4% bovine serum albumin (Sigma) and 4 ug/ml insulin (Sigma) [19]. Fresh medium was supplemented every 2 days. The mammospheres were photographed and counted at day 14. For secondary mammosphere formation, the primary mammospheres were dissociated into single cells, and cultured on 48-well ultra-low-attachment plates usng mammosphere culture medium for another 10 days.

### Side population analysi*s* and CD24^-^CD44^+^ assay

Side population (SP) analysis was performed according to the standard method, as previously described [20]. Briefly, cells were resuspended at 1 × 10^6^ cells per milliliter in DMEM containing 2% FBS, and incubated with 5 μg/ml Hoechst 33342 (Sigma-Aldrich) for 90 minutes at 37 °C with gentle inversion per 15 minutes. Samples treated with 100 M verapamil (Sigma-Aldrich) to block the efflux of the dye served as a negative control. Cells were rinsed with ice cold PBS and then subjected to flow cytometric analysis. For the CD24^-^CD44^+^ assay, conjugated mouse anti-human antibodies phycoerythrin (PE) anti-CD24 and fluorescein isothiocyanate (FITC) anti-CD44 were purchased from BD Pharmingen. Cells were dissociated into single cells using 0.05% trypsin/EDTA (GIBCO), and 1 × 10^6^ cells were resuspended in 200 ul PBS plus 2% FBS, and stained with antibodies for 30 minutes on ice; 4',6-diamidino-2-phenylindole (DAPI; Sigma) was used to identify live or dead cells.

### Wound healing assay and invasion assay

Cells were seeded on 6-well plates, and grown to a confluent monolayer. Streaks were created using a pipette tip. Progression of migration was observed and photographed at initiation time and at 12 hours and 24 hours after wounding. The cell invasion assay was carried out using a Transwell chambers (BD) assay. Cells were suspended in DMEM without serum and plated on the top side of the polycarbonate Transwell filter coated with Matrigel (BD), and the bottom chamber was filled with DMEM supplemented with 10% FBS. After 24 hours of incubation, the non-invasive cells in the top chamber were removed using cotton swabs. The invaded cells were fixed with 4% paraformaldehyde solution and stained with 0.1% crystal violet. Cells were photographed and quantified by counting the cell number in five random fields.

### Soft agar growth assay

The soft agar growth assay was performed according to a standard method, as described previously [21].

### Lucriferase reporter assay

Cells were seeded in triplicate in 24-well plates and allowed to settle for 24 hours. Myc luciferase reporter plasmid pGMMyc-Lu (Genomeditech) plus 1 ng pRL-TK Renilla plasmid were transfected into the cells using Lipofectamine 2000 Reagent (Invitrogen, Carlsbad, CA, USA). After 48-hour transfection, cells were lysed and assayed for luciferase activity using Dual-Luciferase Reporter Assay kit (Promega) according to the manufacturer’s instructions.

### Animal studies

The 6-week-old female nude mice were purchased from Vital River, Beijing. MCF-7 cells at limiting dilution (1 × 10^5^, 1 × 10^4^ and 1 × 10^3^) were injected with Matrigel into the mammary fat pads of the mice. The mice were supplemented with estradiol pellets (0.72 mg, released over 60 days) (Innovative Research of America, Sarasota, FL, USA). Tumor volume was measured every 3 days using a Vernier caliper and calculated according to the formula: Length × Width^2^/2. The mice killed after 30 days, and the tumors were excised and photographed.

### Statistical analysis

Statistical analysis was carried out using the SPSS 17.0 (SPSS Inc.). The results are presented as the mean ± standard deviation for at least three repeated individual experiments for each group. The Student *t* test (two-tailed) was used to statistically analyze the significance of differences between individual groups. Survival curves were plotted by the Kaplan-Meier method and compared by the log-rank test. *P* values <0.05 were considered statistically significant in all cases.

## Results

### Upregulation of TIM is correlated with poor prognosis in breast cancer

**Fig. 1:**
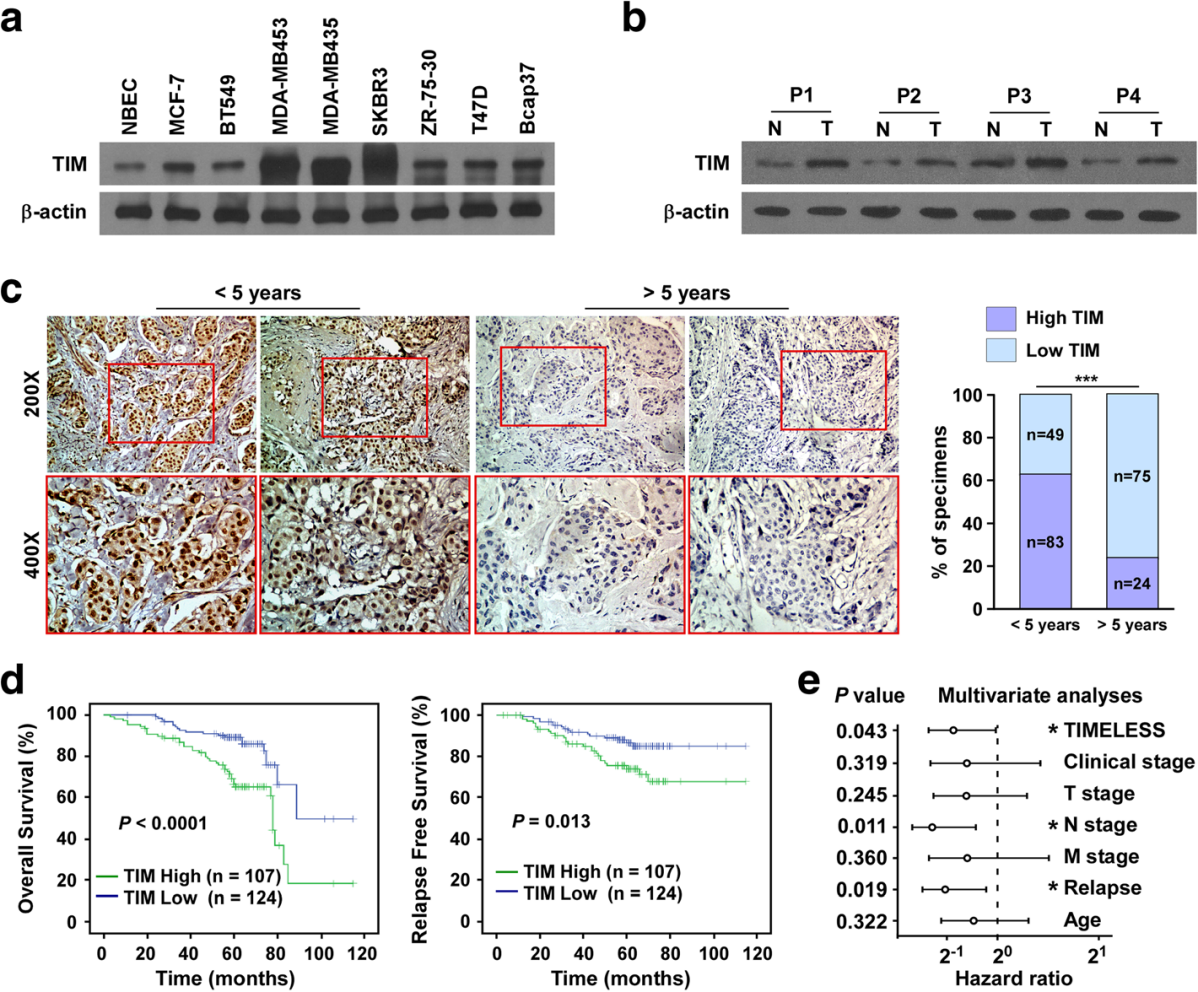
High expression of TIM predicts poor prognosis in patients with breast cancer. **a** TIM expression in normal breast epithelial cells (*NBECs*) and breast cancer cells determined by western blot. **b** TIM expression in four paired primary breast cancer tissue samples (*T*) and matched normal breast tissue samples (*N*) determined by western blot. β-actin was used as a loading control. **c** Immunohistochemistry (IHC) staining of TIM expression in breast cancer tissue came from patients with poor prognosis (less than 5-year survival) and good prognosis (more than 5-year survival). Representative photographs of IHC analysis of breast cancer tissue (*left panel*) and association between TIM expression and prognosis (*right panel*). ***P < 0.001. **d** Kaplan-Meier survival curves indicating the overall survival and relapse-free survival in patients with breast cancer who had high or low protein levels of TIM. **e** Multivariate analysis of clinical factors using a Cox regression model. *T stage* tumor stage, *N stage* nodal stage, *M stage* metastatic stage

To investigate the role of TIM in the progression of breast cancer, we first determined TIM expression in eight breast cancer cell lines. Western blot and real-time-PCR analysis showed that TIM was significantly elevated in breast cancer cells compared with normal breast epithelial cells (NBECs; Fig. 1a-b and Additional file 2: Figure S1A). We further analyzed the expression of TIM mRNA expression in 1102 breast cancer tissue samples and 113 normal breast tissue samples from The Cancer Genome Atlas (TCGA) Data Portal. TIM was significantly upregulated in breast cancer tissue compared to the normal tissue (Additional file 2: Figure S1B-C). Taken together, these results suggest TIM is upregulated in breast cancer.

We next evaluated whether upregulation of TIM was of clinical significance in breast cancer. TIM protein expression was examined in 231 paraffin-embedded, archived breast cancer specimens using immunohistochemistry staining. As shown in Fig. 1c, TIM protein was mainly localized in the nucleus, and primary breast cancer tissues came from patients with poor prognosis as identified by less than 5-year survival, had high TIM expression compared to those coming from patients with good prognosis as identified by more than 5-year survival. Moreover, patients with high levels of TIM expression had shorter overall survival and relapse-free survival (Fig. 1d). Furthermore, multivariate analysis revealed that TIM expression could serve as an independent prognostic marker for breast cancer (Fig. 1e). Consistently, data from Kaplan-Meier Plotter showed that higher expression of TIM was significantly correlated with shorter overall survival, relapse-free survival and distant metastasis-free survival (Additional file 2: Figure S1D). Collectively, these results suggest that there is correlation between high expression of TIM and poor outcome in patients with breast cancer.

### TIM contributes to the expansion of the breast CSC population

**Fig. 2:**
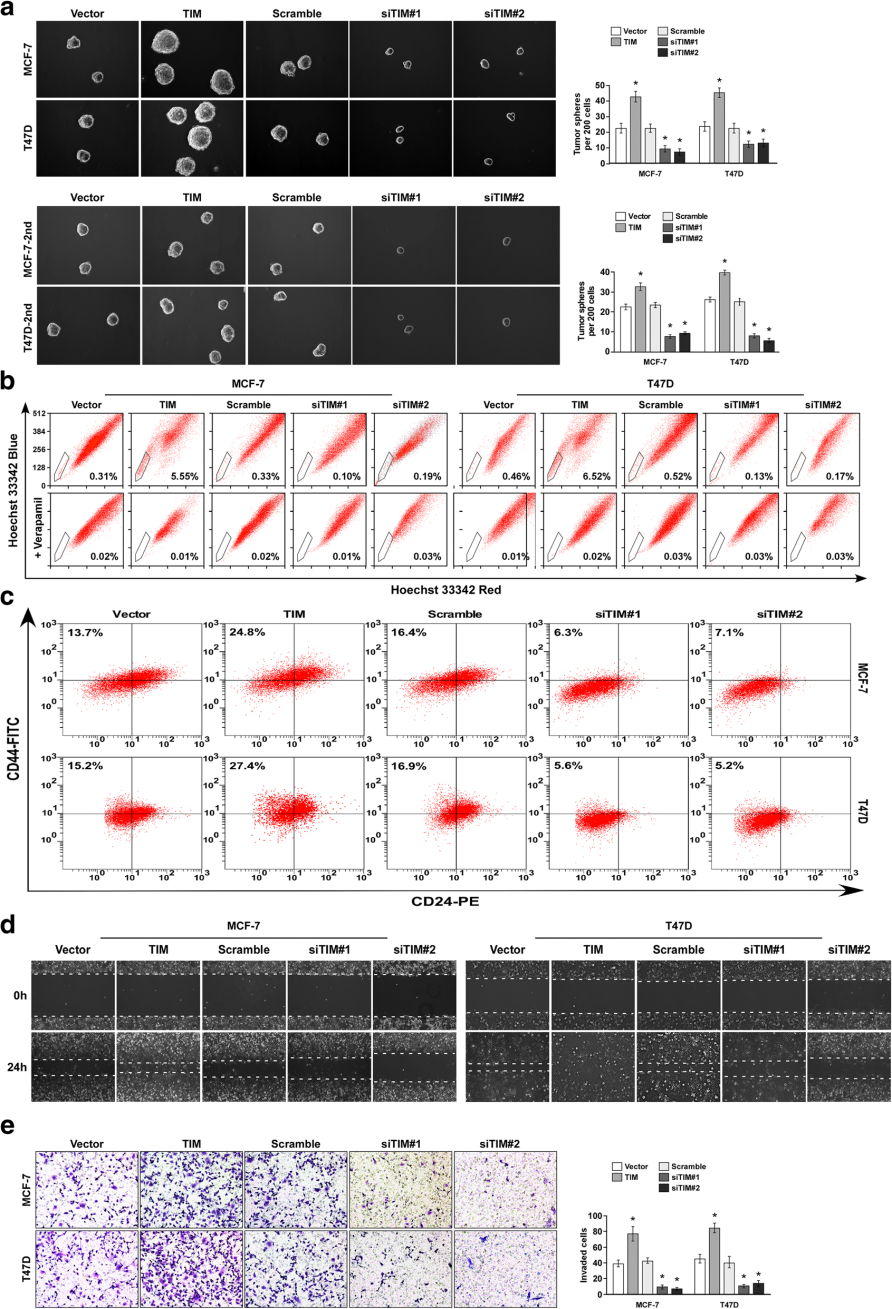
TIM contributes to the expansion of cancer stem cells and cell migration and invasion of breast cancer. **a** Primary and secondary mammosphere formation analysis of MCF-7 and T47D with TIM overexpression or knockdown (*left panel*) and quantification of mammosphere number (*right panel*). Representative photographs of primary mammosphere and mammosphere number (*upper*). Representative photographs of secondary mammosphere and mammosphere number (*bottom*). *P < 0.05. **b** Flow cytometry analysis of side population (SP) cell proportion in the indicated cells. **c** Flow cytometry analysis of CD24^-^CD44^+^ subpopulation in indicated cells. **d** Wound healing assays showing the migratory abilities of the indicated cells. **e** Transwell assays showing the invasive ability of the indicated cells (*left panel*) and quantification of invaded cell number (*right panel*). *P < 0.05. Each *bar* represents mean ± SD of three independent experiments

As CSCs have been implicated with poor clinical outcome in breast cancer [4], we next investigated the role of TIM in the maintenance of CSC-like properties. We first established stable TIM overexpressing or knockdown cell lines in MCF-7 and T47D breast cancer cells. The primary mammosphere formation assay showed that TIM overexpression significantly increased both the number and volume of mammospheres, whereas TIM knockdown reduced both the number and volume of mammospheres, as compared to vector controls. There was a similar result in secondary tumor spheres (Fig. 2a). Moreover, SP analysis demonstrated that overexpression of TIM drastically increased the percentage of SP cells, while knockdown of TIM decreased the percentage of SP cells in MCF-7 and T47D cells (Fig. 2b).

CD24^-^CD44^+^ is a marker for breast cancer CSC [22]; we found that TIM overexpression expanded the CD24^-^CD44^+^ subpopulation and its knockdown reduced the CD24^-^CD44^+^subpopulation (Fig. 2c). Furthermore, through analysis of TIM expression and stem cell-regulated gene signature via gene set enrichment analysis (GSEA) in published expression profiles from patients with breast cancer, we found that there was positive correlation between TIM levels in breast tumors and the embryonic stem cell core signature (Additional file 3: Figure S2A). Taken together, these results suggest that overexpression of TIM contributes to the expansion of CSCs in breast cancer.

The GSEA analysis also revealed that there was positive correlation between TIM levels and the invasive breast cancer and the metastasis gene signatures, indicating that TIM might play a role in breast cancer invasion and metastasis (Additional file 3: Figure S2B and C). The effects of TIM on the invasion and migration of breast cancer cells were further examined using the Transwell invasion assay and wound healing assay. Overexpression of TIM enhanced, while knockdown of TIM reduced the invasive and migratory abilities of MCF-7 and T47D cells, as compared with vector controls (Fig. 2d and e). Collectively, these results suggest that TIM promotes the CSC population and the invasion and migration of breast cancer cells in vitro.

### TIM promotes tumorigenicity of breast cancer cells in vivo

**Fig. 3:**
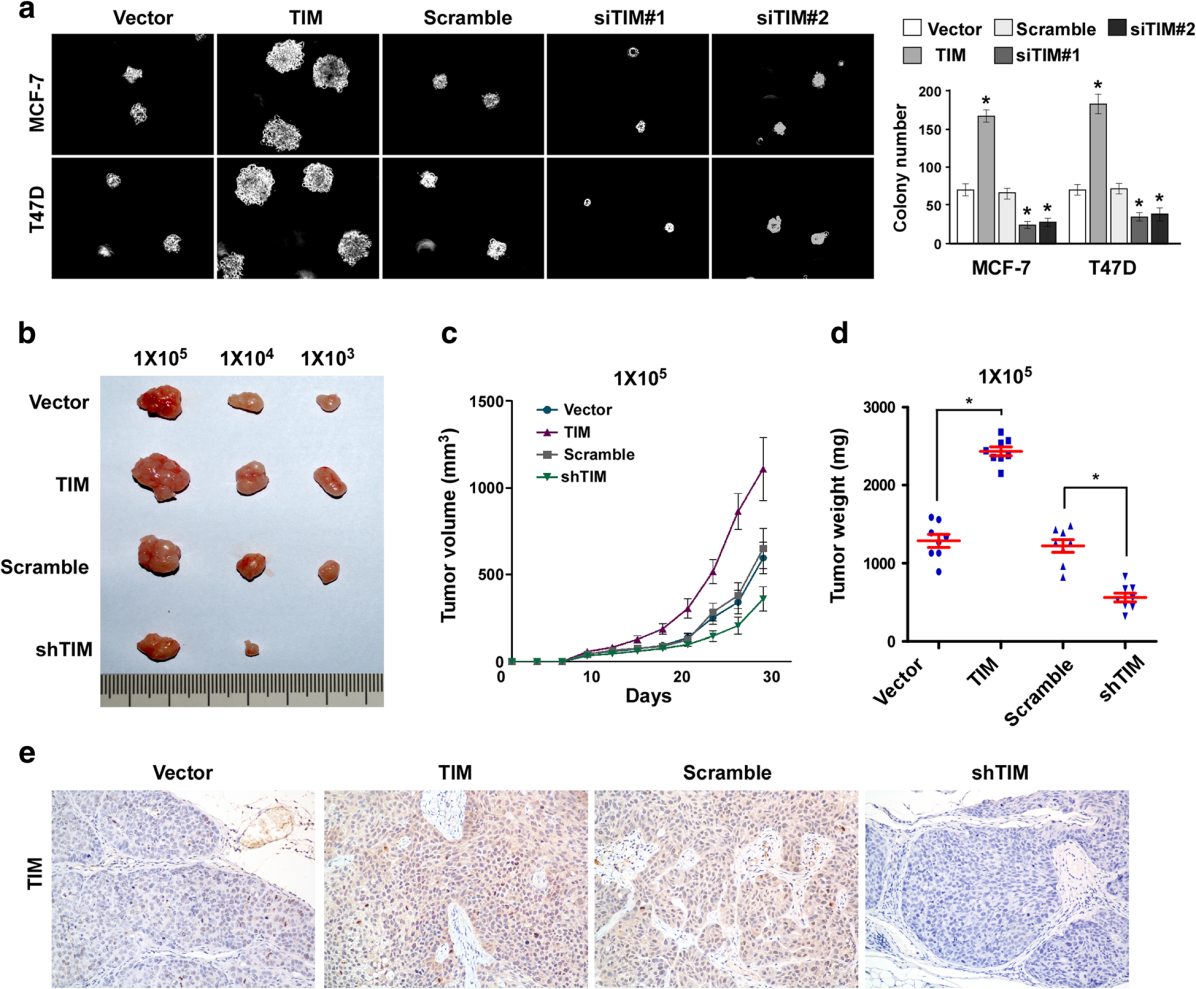
TIM enhances the tumorigenicity of breast cancer cells in vivo. **a** Soft agar growth analysis of indicated cells with TIM overexpression or knockdown (*left panel*) and quantification of colony number (*right panel*). *P < 0.05. **b**-**d** In vivo tumorigenesis assay of MCF-7 cells with TIM overexpression or knockdown. Representative images of the tumors in each group **b**. Growth curves (**c**) and tumor weight (**d**) for tumor formation when different numbers of the indicated cells were injected. Mean tumor volumes are plotted. **e** Immunohistochemistry staining of the xenograft tumor by anti-TIM antibody. Representative photographs are shown

To determine the effect of TIM on the tumorigenic capacity of breast cancer cells, we first examined the anchor-independent growth ability of cells upon manipulation of TIM expression. The soft agar assay showed that overexpression of TIM promoted cell growth and formed more and larger colonies in MCF-7 and T47D cells, as compared with vector controls (Fig. 3a). On the contrary, knockdown of TIM reduced this growth ability (Fig. 3a). We next performed a limiting-dilution transplantation assay to evaluate the in vivo effect of TIM on tumorigenicity of MCF-7 cells. Tumors formed by TIM-overexpressing cells were much larger than those formed by vector control cells, while tumors generated by the TIM-silenced cells were much smaller (Fig. 3b-e). Notably, when 1 × 10^3^ cells with TIM knockdown were injected into nude mice, tumors were not initiated (Fig. 3b). Taken together, these results suggest that TIM promotes tumorigenicity of breast cancer cells in vivo.

### TIM promotes the activation of MYC

**Fig. 4:**
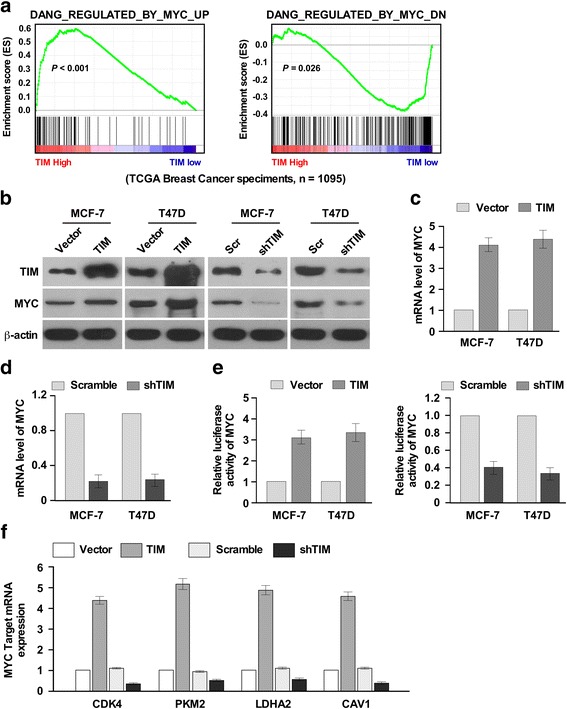
MYC is the downstream effector molecule of TIM. **a** Gene set enrichment analysis showing that TIM expression positively correlated with MYC-regulated gene signatures in The Cancer Genome Atlas (*TCGA*) dataset. **b** Western blot analysis of protein levels of MYC in MCF-7 and T47D with TIM overexpression or knockdown. **c**, **d** Real-time PCR analysis of the mRNA levels of MYC in MCF-7 and T47D with TIM overexpression (**c**) or knockdown (**d**). **e** Luciferase analysis indicating the transactivity of MYC in MCF-7 and T47D with TIM overexpression or knockdown. *P < 0.05. Each *bar* represents mean ± SD of three independent experiments. **f** Real-time PCR analysis of the mRNA levels of MYC target genes including *CDK4*, *PKM2*, *LDHA2* and *CAV1* in MCF-7 and T47D with TIM overexpression or knockdown

We further investigated the mechanism by which TIM enhances the CSC property of breast cancer cells. GSEA analysis on the TCGA data set showed that TIM expression was positively correlated with gene signature upregulated by MYC and was inversely correlated with gene signature downregulated by MYC (Fig. 4a), indicating that TIM might regulate the activation of MYC. Moreover, western blot and real-time PCR analyses showed that overexpression of TIM significantly increased, while knockdown of TIM decreased the expression level of MYC in MCF-7 and T47D cells (Fig. 4b-d). Furthermore, overexpression of TIM significantly enhanced, while silencing of TIM reduced the transcriptional activity of MYC and the mRNA levels of several MYC target genes including *CDK*, *PKM*, *LDHA2* and *CAV1* (Fig. 4e–f). Collectively, these results suggest that TIM promotes the activation of MYC.

#### MYC is required for TIM-mediated CSC self-renewal and cell invasion of breast cancer

**Fig. 5:**
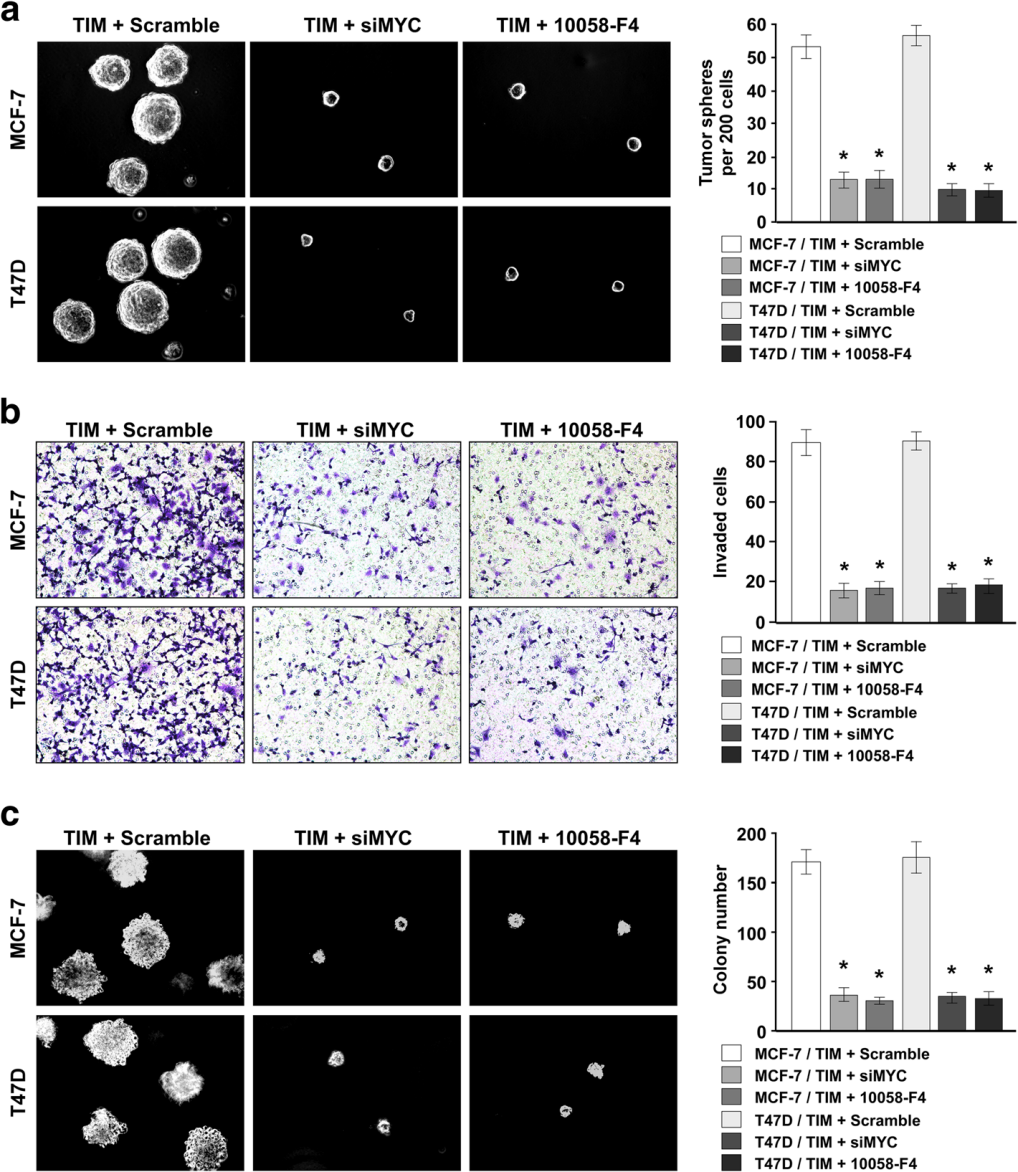
Inhibition of MYC abrogates the phenotype caused by TIM overexpression. **a** Mammosphere formation analysis of cells with indicated treatment (*left panel*) and quantification of mammosphere number (*right panel*). *P < 0.05. **b** Transwell analysis of cells with indicated treatment (*left panel*) and quantification of invaded cell number (*right panel*). *P < 0.05. **c** Soft agar growth analysis of cells with the indicated treatment (*left panel*) and quantification of colony number (*right panel*). *P < 0.05. Each *bar* represents mean ± SD of three independent experiments

Next, we investigated whether TIM-mediated CSC self-renewal and cell invasion are dependent on MYC activation. Silencing of MYC or inhibiting MYC activation using an inhibitor (10058-F4) in TIM-overexpressing cells significantly suppressed the self-renewal capability of CSC-like cells and cell invasion, as determined by mammosphere formation and Transwell assays (Fig. 5a, b). Moreover, downregulation or inhibition of MYC significantly reduced cell growth ability in soft agar in TIM-overexpressing cells (Fig. 5c). Together, our data suggest that MYC is required for TIM-mediated CSC self-renewal and cell invasion in breast cancer.

## Discussion

Here, we studied the role of TIM in the progression of breast cancer. We reported that TIM functions as an oncogenic protein to promote tumorigenicity, migration and invasion in breast cancer. TIM exerts its function in breast cancer by upregulation of the oncogene MYC. These findings suggest a critical role for TIM in promoting development and progression of breast cancer.

It is reported that TIM is upregulated in patients with relapse of estrogen receptor (ER)α-positive breast cancer, who have been treated with tamoxifen, and that TIM is overexpressed in OH-Tam-resistant breast cancer cells [23]. Tamoxifen-resistant cells have much stronger invasive, migratory and mammosphere forming abilities with increased percentage of CD24^-^CD44^+^ marked breast CSCs [24–26]. Consistently, in our study, we demonstrated that TIM significantly increased mammosphere formation and the proportion of SP cells, suggesting that TIM represents a positive regulator of breast CSC-like property and enhances the chemo-resistance of breast cancer cells. Moreover, we also found that TIM enhanced the invasive and migratory abilities of breast cancer cells. Our study provides evidence suggesting that TIM may be responsible for the increased invasive, migratory and mammosphere forming abilities of tamoxifen-resistant cells.

MYC is a well-known oncogene; it plays a pivotal role in cell growth, tumorigenesis and stem cell self-renewal [27, 28]. MYC is identified as one of the four reprogram factors including Oct4, Sox2 and KLF4 that can dedifferentiate fibroblasts to pluripotent stem cells [29, 30]. It is reported that MYC induces polycomb proteins Bmi-1 and EZH2 to maintain stem cell pluripotency [31–33]. In our study, we found that TIM upregulated the expression of MYC, thus to promote breast CSC self-renewal. Our findings revealed a novel function of TIM in the regulation of MYC and CSC properties. Moreover, it is also well-documented that MYC represses genes involved in cell-cell contact, to induce epithelial-mesenchymal transition (EMT) and enhance metastasis of cancer cells [34]. MYC can regulate miR-9, which targets E-cadherin, and transactivates Bmi-1 to facilitate EMT and metastasis [35, 36]. We demonstrated that TIM increased the invasive and migratory abilities of breast cancer cells at least partially through regulation of MYC expression. Suppression of MYC compromised the TIM-induced invasiveness and migration of breast cancer cells. Taken together, our data suggest that MYC is the critical downstream effector in TIM-mediated stem cell self-renewal, cell migration and invasion in breast cancer.

## Conclusions

In summary, this study provides the first evidence that TIM promotes aggressive progression of breast cancer through upregulation of MYC, contributing to the poor prognosis of patients with breast cancer. Our study elucidates the precise role of TIM in the progression of breast cancer, and may help to identify novel effective therapeutic strategies for human breast cancer.

## Additional files

Additional file 1: Table S1. The clinicopathological characteristics of breast cancer patient samples (DOCX 14 kb)

Additional file 2: Figure S1. High expression of TIM is correlated with poor prognosis of patients with breast cancer. **A** TIM expression in normal breast epithelial cells NBECs and breast cancer cells determined by real-time PCR. Each *bar* represents mean ± SD of three independent experiments. **B** and **C** mRNA level of TIM expression analysis in normal breast tissues and primary breast cancer tissues using the data downloaded from TCGA database. **P < 0.01. **D** Kaplan-Meier survival curves indicating the overall survival, relapse-free survival and distant metastasis-free survival of breast cancer patients with low or high levels of TIM from the Kaplan Meier Plotter website (TIF 257 kb)

Additional file 3: Figure S2. GSEA analysis showing that TIM expression positively correlated with cancer stem cell, invasive and metastasis gene signatures in TCGA dataset (TIF 290 kb)

## Abbreviations

CSCs: Cancer stem cells
Ct: Cycle threshold
DAPI: 4',6-Diamidino-2-phenylindole
DMEM: Dulbecco’s modified Eagle’s medium
EEF1A2: Eukaryotic elongation factor 1A2
EGF: Epidermal growth factor
EMT: Epithelial-mesenchymal transition
FBS: Fetal bovine serum
FITC: Fluorescein isothiocyanate
GAPDH: Glyceraldehyde-3-phosphate dehydrogenase
GSEA: Gene set enrichment analysis
IHC: Immunohistochemistry
NBEC: Normal breast epithelial cell line
PBS: Phosphate-buffered saline
PCR: Polymerase chain reaction
PE: Phycoerythrin
siRNA: Small interference RNA
SP: Side population
TGCA: The Cancer Genome Atlas
TIM: TIMELESS
Tipin: TIM-interacting protein
