# Supplementary material for: TIMELESS contributes to the progression of breast cancer through activation of MYC
Source: Breast Cancer Res. 2017 May 2;19:53. doi: 10.1186/s13058-017-0838-1 (PMC5414141; doi:10.1186/s13058-017-0838-1)
Supplement: Supplementary file 1 — The clinicopathological characteristics of breast cancer patient samples (DOCX 14 kb) [file 13058_2017_838_MOESM1_ESM.docx]

**Table S1. The clinicopathological characteristics of breast cancer patient samples.**

|  | **Number of cases** |
| --- | --- |
| **Age(years)** |  |
| > 45 | 102 |
| ≤ 45 | 129 |
| **Clinical Stage** |  |
| I | 40 |
| IIa | 67 |
| IIb | 30 |
| IId | 6 |
| IIIa | 39 |
| IIIb | 14 |
| IIIc | 15 |
| IV | 20 |
| **T classification** |  |
| T1 | 67 |
| T2 | 120 |
| T3 | 31 |
| T4 | 13 |
| **N classification** |  |
| N0 | 94 |
| N1 | 70 |
| N2 | 41 |
| N3 | 26 |
| **M classification** |  |
| Yes | 20 |
| No | 211 |
| **ER** |  |
| Negative | 114 |
| + | 42 |
| ++ | 22 |
| +++ | 53 |
| **PR** |  |
| Negative | 106 |
| + | 40 |
| ++ | 37 |
| +++ | 48 |
| **HerB2** |  |
| Negative | 114 |
| + | 29 |
| ++ | 25 |
| +++ | 63 |
| **Radiation treatment** |  |
| Yes | 82 |
| No | 56 |
| Unknown | 93 |
| **Pharmaceutical treatment** |  |
| Yes | 138 |
| No | 28 |
| Unknown | 65 |
| **Recrudescence** |  |
| Yes | 42 |
| No | 189 |
| **Survive or Mortality** |  |
| Survive | 170 |
| Mortality | 61 |
