# Supplementary figures and images for: TIMELESS contributes to the progression of breast cancer through activation of MYC
Source: Breast Cancer Res. 2017 May 2;19:53. doi: 10.1186/s13058-017-0838-1 (PMC5414141; doi:10.1186/s13058-017-0838-1)

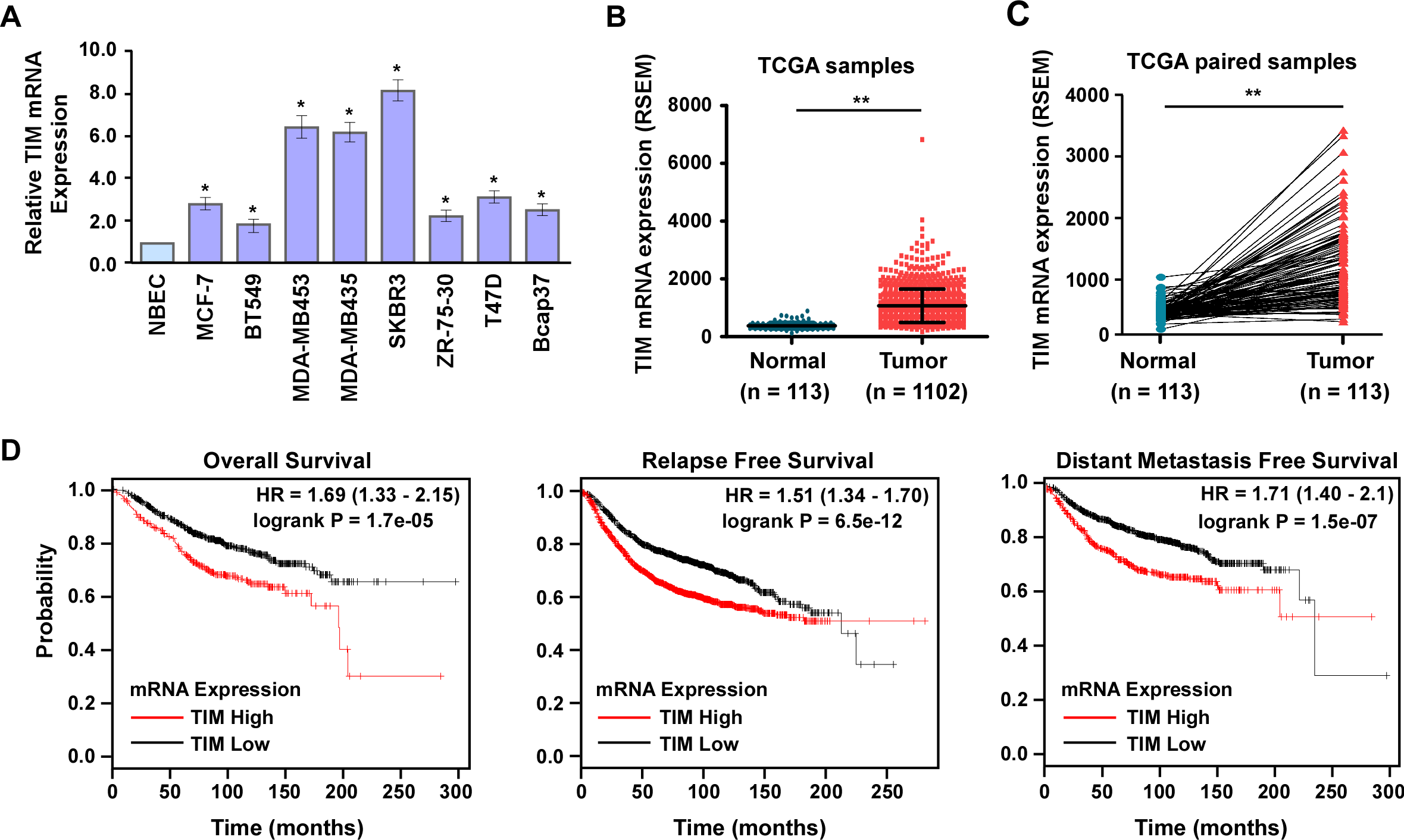

Supplement: Supplementary file 2 — High expression of TIM is correlated with poor prognosis of patients with breast cancer. A TIM expression in normal breast epithelial cells NBECs and breast cancer cells determined by real-time PCR. Each bar represents mean ± SD of three independent experiments. B and C mRNA level of TIM expression analysis in normal breast tissues and primary breast cancer tissues using the data downloaded from TCGA database. **P < 0.01. D Kaplan-Meier survival curves indicating the overall survival, relapse-free survival and distant metastasis-free survival of breast cancer patients with low or high levels of TIM from the Kaplan Meier Plotter website (TIF 257 kb) [file 13058_2017_838_MOESM2_ESM.tif]

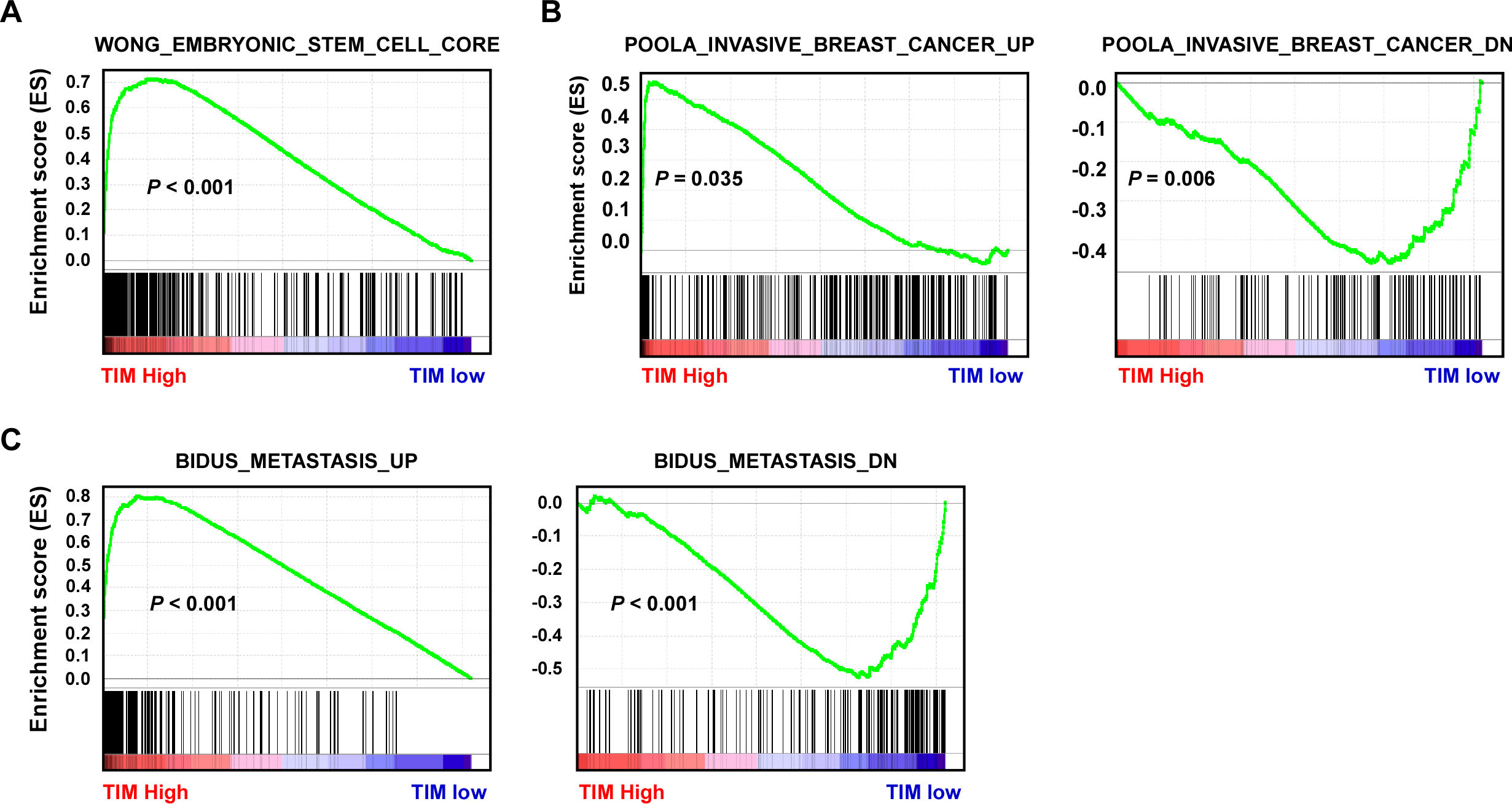

Supplement: Supplementary file 3 — GSEA analysis showing that TIM expression positively correlated with cancer stem cell, invasive and metastasis gene signatures in TCGA dataset (TIF 290 kb) [file 13058_2017_838_MOESM3_ESM.tif]
